# Critical Management of Septic Orthopedic Patients: The Impact of Intensive Care on Survival and Recovery

**DOI:** 10.3390/life15040674

**Published:** 2025-04-21

**Authors:** Angelica Bratu, Catalin Cirstoiu, Mihnea Ioan Gabriel Popa, Mihai Popescu, Oana Clementina Dumitrascu, Mihaela Agapie, Carmen Orban

**Affiliations:** 1 Department of Anesthesiology and Intensive Care, Emergency University Hospital Bucharest, 050098 Bucharest, Romania; angibratu@yahoo.com (A.B.); mihai.popescu@umfcd.ro (M.P.); oanadumit@gmail.com (O.C.D.); agapiemili@yahoo.com (M.A.); carmen.orban@umfcd.ro (C.O.); 2 Department of Orthopedics and Traumatology, “Carol Davila” University of Medicine and Pharmacy, 050474 Bucharest, Romania; 3 Department of Orthopedics and Traumatology, Emergency University Hospital Bucharest, 050098 Bucharest, Romania; 4 Department of Anesthesiology and Intensive Care, “Carol Davila” University of Medicine and Pharmacy, 050474 Bucharest, Romania

**Keywords:** orthopedic infections, intensive care unit, periprosthetic joint infections, antimicrobial therapy, multidisciplinary care

## Abstract

The management of septic orthopedic patients, particularly those with periprosthetic joint infections (PJIs) and trauma-related sepsis, remains a significant clinical challenge. This retrospective cohort study evaluated 27 patients admitted to the Intensive Care Unit (ICU) at the Emergency University Hospital in Bucharest between 2021 and 2024. Patients presented with either PJIs or polytrauma-related infections requiring critical care interventions. The PJI-TNM classification system was employed to assess infection complexity, comorbidities, and implant stability. Therapeutic strategies included one- or two-stage revision surgeries and targeted antimicrobial therapy, including the use of antibiotic-impregnated calcium sulfate beads. Infection resolution was achieved in 85.2% of patients, with a mean ICU stay of 13 days. The overall ICU mortality rate was 11%, with two deaths occurring within the first 30 days of admission. Elevated SOFA scores (≥10) and poor glycemic control (HbA1c > 8.5%) were significantly associated with prolonged ICU stays and higher complication rates. Statistical analysis revealed significant differences in CRP normalization and bone healing times across glycemic control groups (*p* < 0.001). Patients requiring mechanical ventilation exhibited longer ICU stays and increased mortality (25%). The PJI-TNM classification showed potential utility for risk stratification and guiding personalized treatment strategies. These findings underscore the importance of multidisciplinary ICU-level care and metabolic control in improving outcomes for septic orthopedic patients. Future multicenter studies are needed to validate these preliminary observations and refine prognostic models for this high-risk population.

## 1. Introduction

In recent years, infections in the field of orthopedic surgery have been an increasing concern, reflecting the increase in hip and knee arthroplasties and the continuous rise of polytraumatized patients requiring surgical interventions for fracture stabilization. Advanced age, associated comorbidities, and the immunosuppressive status often associated with elderly patients represent significant risk factors in the development of sepsis associated with orthopedic implants. Conversely, the polytraumatized patient, frequently presenting with open fractures and associated traumatic/hemorrhagic shock, is also highly susceptible to the development of subsequent sepsis related to the orthopedic implant. Sepsis in the field of orthopedic surgery has significant implications for morbidity and mortality, the length of hospitalization, and, consequently, the costs of medical care. It also affects the incidence of complications or decompensation of pre-existing pathologies [1,2].

Multidisciplinary management (orthopedist, intensivist, infections, etc.) is mandatory and prevention is crucial. This necessitates the identification of associated risk factors and meticulous preoperative preparation in accordance with current guidelines, surgical procedures conducted under strict aseptic and antisepsis conditions, and careful postoperative surveillance for the early identification of the development of a possible septic process [3,4,5].

Pathogenic bacteria use the surface of the orthopedic implant as a medium for colonization, resulting in the formation of a biofilm that facilitates the propagation and amplification of the septic process. In the majority of cases, the microorganisms involved are Gram-positive cocci, including Staphylococcus aureus, Staphylococcus epidermidis, and Streptococcus pyogenes. However, Gram-negative bacteria, such as Pseudomonas aeruginosa, and fungi, including Candida albicans and other species, may also be involved [6,7,8]. The breach of mechanical barriers, local wound contamination, and invasive interventions serve to significantly heighten the risk of infection in trauma patients [9,10]. The findings of our study highlight the importance of a multidisciplinary approach, and the rapid implementation of vital functions support measures alongside surgical treatment. This approach is essential for improving the outcome of patients with implant-related orthopedic sepsis [3,4,6].

Periprosthetic joint infections represent a significant complication in orthopedic patients, particularly following joint arthroplasty, especially in patients with comorbidities or immunosuppression [11,12,13]. These infections impose a considerable economic burden, longer hospitalizations, prolonged antibiotic treatment, revision procedures, and increasing the risk of complications and admission to the intensive care unit (ICU) due to multi-organ failure. As the global population continues to age and the prevalence of joint replacement procedures increases, it is anticipated that the frequency of prosthetic joint infections (PJIs) will rise, consequently increasing the number of patients requiring critical care. Research findings indicate that one-year mortality rates post-PJI of the hip reach 13.6%, rising to 25.6% within five years. These statistics are comparable to or exceed those observed in various malignancies. The PJI-TNM categorization system, analogous to the oncological TNM staging system, provides a valuable tool for assessing the complexity of PJIs and for guiding the development of personalized treatment plans. The PJI-TNM system enables physicians to stratify at-risk patients and personalize treatments by evaluating characteristics such as implant stability, biofilm formation, and patient comorbidities, which is particularly crucial for individuals who may require intensive care interventions [14,15,16].

Septic orthopedic infections represent a significant complication, particularly in individuals with trauma or subsequent to arthroplasty. Such infections have the potential to rapidly advance to systemic sepsis, necessitating prompt management in the ICU. The transition from a localized infection to systemic sepsis is regulated by intricate physiological mechanisms, particularly involving systemic inflammatory response syndrome (SIRS) and compensatory anti-inflammatory response syndrome (CARS) [8,9]. CARS may inhibit the immunological response, rendering patients vulnerable to opportunistic infections and thus exacerbating their disease [8,14,15].

The management of septic orthopedic patients in the ICU is a complex process that requires advanced support for organ dysfunctions and antibiotic treatment. It is often necessary to provide ventilatory support to patients with sepsis or septic shock due to acute respiratory distress syndrome (ARDS). Mechanical ventilation, in conjunction with hemodynamic monitoring, is vital for maintaining tissue perfusion and oxygenation, particularly in cases of septic shock [13,16,17]. The probability of multi-organ failure is increased in these patients, with frequent complications affecting the kidneys, liver, and cardiovascular systems. In order to stabilize the patient, aggressive fluid resuscitation, vasopressors, and, in critical instances, continuous renal replacement therapy (CRRT) is often required [1,15,18]. The cytokine storm associated with sepsis serves to exacerbate these symptoms, and in the absence of appropriate management, may result in irreparable organ damage. Furthermore, it is of the utmost importance to regulate glucose levels carefully in order to prevent stress-induced hyperglycemia. However, this equilibrium must be closely monitored to avoid the risk of hypoglycemia, which could potentially exacerbate the situation [1,10,15,19].

Antimicrobial therapy is of paramount importance in the management of septic illnesses. In the initial stages of treatment, broad-spectrum antibiotics are administered empirically, with modifications being made according to the results of culture tests. The selection of antibiotics must take into account the potential for antimicrobial resistance (AMR), particularly in ICU patients who are subjected to prolonged antibiotic regimens. The formation of biofilms by bacteria complicates the treatment of prosthetic joint infections, as the antibiotic potency is diminished. In many cases, surgical procedures, such as debridement or excision of the infected prosthesis, are required to completely eliminate the infection. In cases of prosthetic joint infection, the use of targeted antibiotics, such as antibiotic-impregnated calcium sulfate beads, may prove an effective additional treatment option [1,3,14]. It is of the utmost importance to implement antibiotic stewardship in the ICU in order to prevent the emergence of antibiotic-resistant pathogens. Patients in the ICU, particularly those with complex illnesses such as PJIs, are at an elevated risk of developing multi-drug-resistant infections due to their prolonged exposure to broad-spectrum antibiotics. The implementation of consistent assessments of antibiotic effectiveness, in conjunction with infection control measures, can serve to mitigate the risk of multi-drug-resistant infections and enhance patient outcomes [1,3,20].

The objective of this study was to evaluate the clinical outcomes, survival rates, and inflammatory response trajectories in septic orthopedic patients requiring ICU-level management, with a specific focus on cases of PJI and polytrauma-related sepsis. The study also aimed to assess the potential prognostic value of early SOFA scoring and the applicability of the PJI-TNM classification system in guiding therapeutic decisions [7,21].

## 2. Materials and Methods

### 2.1. Study Design and Population

This retrospective cohort study analyzed data from 27 patients admitted to the ICU at the Emergency University Hospital, Bucharest, between 1 January 2021 and 31 December 2024. The patients in question were referred to by the Department of Orthopedics and Traumatology. Patients were classified into two primary categories based on their primary diagnosis: (1) Polytrauma-related sepsis, referring to patients with severe traumatic injuries and associated infections, and (2) Periprosthetic Joint Infections, referring to patients with infections localized to orthopedic implants or prosthetic joints. While these categories were analyzed separately for specific outcomes, it is important to note that some patients presented overlapping characteristics, such as trauma-induced prosthetic infections. Overlapping cases were considered based on their primary diagnosis, as determined by clinical judgment and multidisciplinary team evaluation. All patients received treatment following a diagnosis of sepsis or septic shock, as defined by Sepsis-3 criteria and the International Consensus on Orthopedic Infections. Ethical approval was obtained from the Institutional Review Board (Ethics Committee) of Spitalul Universitar de Urgență București (protocol code 63857), in accordance with national and international ethical guidelines. Informed consent was waived due to the retrospective nature of the study; however, all patient data were anonymized to maintain confidentiality. While the sample size (n = 27) may appear limited, it reflects a highly selective cohort requiring ICU-level interventions, making this a clinically relevant population. Given the rarity of orthopedic infections requiring critical care, our study provides a foundational analysis that can be expanded in future multicenter research. Terminology Clarification: For the purposes of this study, the following definitions were applied to ensure terminological consistency: Polytrauma-related sepsis refers to systemic infection arising in patients with multiple traumatic injuries, often involving open fractures or surgical wounds, with sepsis secondary to microbial invasion in the context of trauma. Trauma-induced prosthetic infections describe cases in which prosthetic joint infections developed subsequent to initial traumatic injury and surgical intervention, usually involving internal fixation or primary arthroplasty. PJIs are defined as infections occurring around a joint prosthesis, as classified by the International Consensus Meeting (ICM) guidelines. Chronic infections are those persisting for more than 4 weeks, typically involving mature biofilm formation, and confirmed by microbiological, radiological, and clinical criteria.

**Figure 1:**
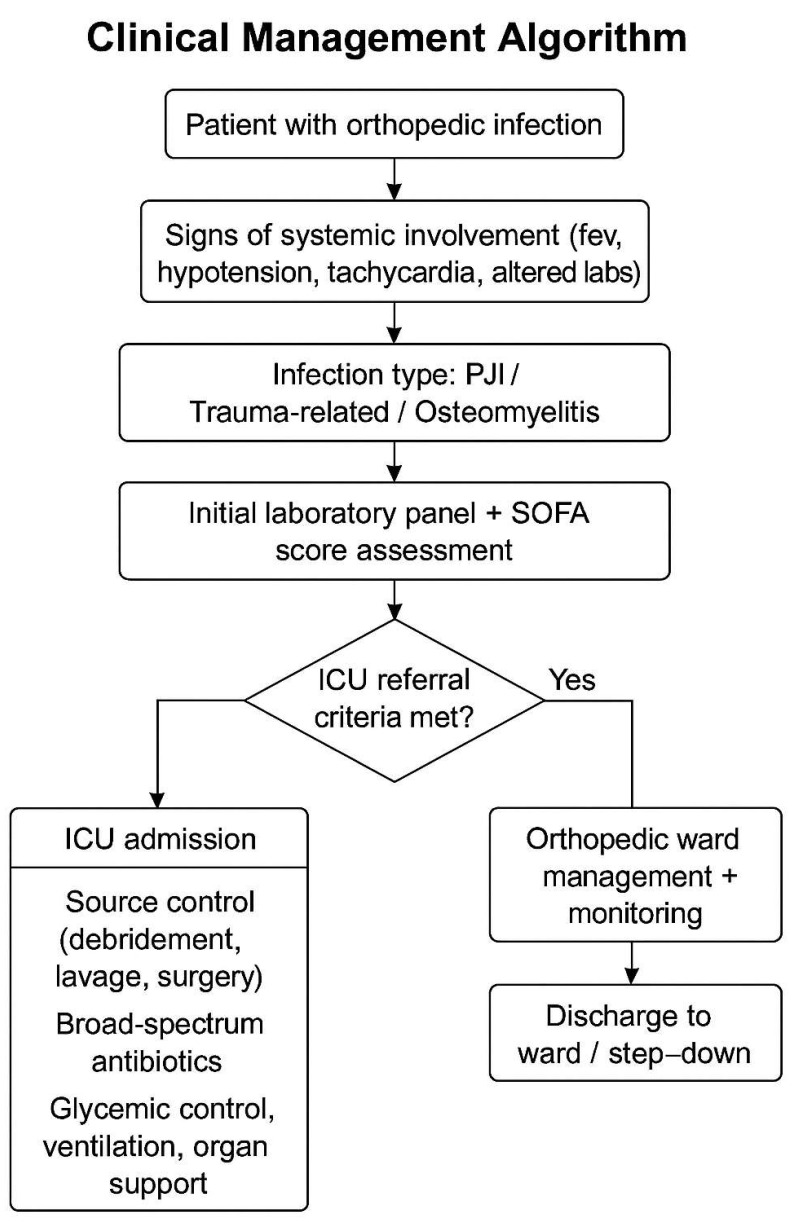
Clinical Management Algorithm for Septic Orthopedic Patients.

A structured multidisciplinary protocol was used for the management of septic orthopedic patients, based on the severity of infection, comorbidities, and presence of systemic decompensation. The algorithm in Figure 1 outlines the clinical decision-making steps from initial presentation to ICU admission, treatment strategy, and follow-up pathway.

The flowchart illustrates the decision-making process used at our institution to evaluate and manage orthopedic patients with systemic infections. ICU referral was based on SOFA scores, laboratory and clinical indicators of sepsis, and the need for advanced organ support. Multidisciplinary treatment included source control, antimicrobial therapy, and metabolic stabilization.

### 2.2. Inclusion and Exclusion Criteria

The following patients will be included in the study: Patients aged 18 years or over who have been admitted to the ICU from the Orthopedic Department for the management of severe sepsis or septic shock; patients with confirmed septic disease related to polytrauma or PJI; patients with microbiologically confirmed bacterial, fungal, or viral pathogens. Patients will be excluded if they have incomplete medical records if they are lost to follow-up; if the infection is unrelated to orthopedic problems; or if the inflammation is due to a non-bacterial, non-fungal, or non-viral source (e.g., autoimmune disorders). Infections were classified as chronic according to the criteria outlined by the International Consensus Meeting on Prosthetic Joint Infection (ICM). Chronic infection was defined as one with persistent symptoms lasting more than 4 weeks, microbiological evidence of mature biofilm formation, and radiological changes suggestive of chronic infection.

The cohort size (n = 27) reflects the strict inclusion criteria applied in this study, focusing exclusively on patients with severe orthopedic infections requiring ICU admission. These cases are inherently rare, as the majority of orthopedic infections are managed outside of critical care settings. Furthermore, the study prioritized patients with either polytrauma-related sepsis or PJIs with systemic involvement, further narrowing the eligible population. Future multicenter investigations could provide additional statistical power to further validate these findings.

### 2.3. Treatment Protocols

The treatment strategies were tailored to the specific characteristics of each patient, with consideration given to the severity and progression of sepsis. All patients received an initial course of broad-spectrum antibiotic therapy upon admission to the intensive care unit, with subsequent adjustments based on culture and extended antibiogram results. The surgical management of PJIs entailed either a one-stage or two-stage revision procedure, contingent on the severity of the infection, the patient’s comorbidities, and the presence of a biofilm.

Periprosthetic Joint Infection Protocol:

- Two-Stage Revision (n = 15): Debridement, implantation of antibiotic-impregnated cement spacers, and subsequent reimplantation after infection clearance.
- One-Stage Revision (n = 3): The immediate removal of the infected prosthesis is followed by the implantation of a replacement prosthesis and the administration of high-dose systemic antibiotics.
- Additional Therapy: In both revision protocols, antibiotic-infused calcium sulfate beads were employed to augment local antibiotic concentrations and target biofilms.

Polytrauma Protocol (n = 9): Patients with septic shock received aggressive fluid resuscitation in accordance with the Surviving Sepsis Campaign Guidelines, vasopressor support, and expeditious fracture stabilization. Ventilatory support was provided as needed for acute respiratory distress syndrome (ARDS).

### 2.4. Data Collection and Study Objectives

The data were recorded from the patients’ observation sheets and comprised a range of information, including demographic data, clinical examinations, laboratory analyses, microbiological results, and surgical interventions.

Mortality was assessed at two-time points: (1) 30-day mortality, referring to deaths occurring within the first 30 days after ICU admission, and (2) overall mortality, referring to deaths occurring at any point during the patient’s ICU stay or follow-up period.

The primary objectives were as follows:

- Achieve remission of the infectious process, which was assessed clinically by the local evolution of the surgical wound and biologically by the attenuation of the systemic inflammatory syndrome (leucocytes count, CRP, IL-6). Microbiological samples (surgical wounds, blood cultures) were also tested for the presence/absence of bacteria.
- Mortality at 30 days

The secondary objectives were as follows:

- The necessity of mechanical ventilation;
- The necessity of CRRT;
- The severity of the disease at the time of ICU admission was evaluated by Sequential Organ Failure Assessment (SOFA).

Mortality data were obtained from hospital records and the institutional electronic patient tracking system.

### 2.5. Statistical Analysis

All statistical analyses were conducted using IBM SPSS Statistics (version 27.0, IBM Corp., Armonk, NY, USA). Continuous variables were expressed as mean ± standard deviation (SD). The normality of data distribution was verified using the Shapiro–Wilk test. Group comparisons between patients with different glycemic control levels (Group A: HbA1c < 7%, Group B: 7–8.5%, Group C: >8.5%) were performed using one-way ANOVA and post-hoc analysis when appropriate. A *p*-value of less than 0.05 was considered statistically significant. Additionally, variations of means were explored descriptively between subgroups. Propensity score analysis was not performed due to sample size limitations, and no multivariate regression models were applied.

### 2.6. Ethical Considerations

This research was conducted in accordance with the ethical principles set forth in the Helsinki Declaration. Prior to the commencement of the study, ethical approval was obtained from the Institutional Review Board (Ethics Committee) of Spitalul Universitar de Urgență București (protocol code 63857, approved on 10 December 2020). In view of the retrospective nature of the data gathering in the study, the necessity for patient permission was waived. All patient information was anonymized, and data were managed in accordance with the General Data Protection Regulation (GDPR) to guarantee the confidentiality and integrity of the data.

## 3. Results

### 3.1. Patient Demographics and Infection Characteristics

From 1 January 2021 to 31 December 2024, 27 patients with severe orthopedic infections were admitted to the ICU at the Emergency University Hospital in Bucharest. The patients exhibited either polytrauma-related sepsis (nine instances) or PJIs (eighteen cases). The study cohort comprised 16 male patients (59%) and 11 female patients (41%), with ages ranging from 42 to 83 years and a mean age of 67.3 years. The gender distribution indicated a higher prevalence of septic orthopedic complications in males, particularly in instances related to trauma.

In order to evaluate the complexity of the infections and the overall health state of the patients, the infections were retrospectively classified using the PJI-TNM (Table 1) classification system to guide treatment planning by stratifying infection severity based on implant stability, biofilm formation, and comorbidities. However, detailed stratification outcomes are not presented here due to data limitations. Future research with larger cohorts may allow a more robust analysis of outcomes based on TNM stratification, providing deeper insights into risk stratification and tailored intervention strategies. The classification revealed that most infections (70%) were chronic, with fully established biofilms impeding the efficacy of the administered therapy. Out of the 27 patients included in the study, 19 (70%) were diagnosed with chronic infections. This classification was based on clinical criteria (persistence of symptoms for more than 4 weeks), microbiological findings (evidence of mature biofilm formation), and radiological signs (suggestive of chronic infection). In prosthetic joint infections, the predominant pathogen was identified as Staphylococcus aureus (including methicillin-resistant strains—20%), followed by Staphylococcus epidermidis (21%), and Candida albicans (5.2%).

Among the 27 patients, 9 were categorized primarily as polytrauma-related sepsis and 18 as PJI. In cases where overlap existed (e.g., polytrauma patients developing PJI post-trauma), classification was based on the primary clinical concern at ICU admission. These overlapping cases were analyzed within their respective primary categories to maintain clarity in outcome comparisons. Future studies with larger cohorts may allow for a more detailed analysis of these overlapping cases. The study population exhibited a high prevalence of comorbidities, including diabetes mellitus (52%), cardiovascular disease (34%), and chronic renal failure (23%). These disorders had a significant impact on treatment outcomes, frequently impeding recovery due to compromised immune function and protracted wound healing. The mean BMI among patients was 29.1 ± 3.8 kg/m^2^. According to WHO classification, 18.5% were of normal weight, 48.1% were overweight, and 33.3% were classified as obese. The PJI-TNM classification system was retrospectively applied to all patients with periprosthetic joint infections (n = 16). Most were staged as T2 (n = 8) or T3 (n = 3), reflecting moderate-to-severe local extension. Comorbidity burden (N stage) was high, with 10 of 16 patients classified as N2. Difficult-to-treat pathogens (MRSA, Candida) led to an M1 classification in 6 cases. The presence of multiple comorbidities increased the likelihood of multi-organ dysfunction and prolonged ICU admissions (Table 2).

In three patients aged 75 years or older with substantial comorbidities, a one-stage revision surgery was conducted due to the elevated mortality risk associated with a two-stage procedure. The patients were administered intensive antibiotic therapy and received antibiotic-impregnated cement spacers. They were also monitored meticulously for any postoperative complications.

Among the polytrauma patients, extensive microbial contamination was observed, with predominant pathogens including MRSA, Pseudomonas aeruginosa, Enterobacter, Klebsiella pneumoniae, and Acinetobacter baumannii MDR. In the acute phase, infections were characterized by high bacterial diversity, making initial pathogen-specific targeting challenges. To address this, a broad-spectrum antibiotic regimen was initiated, later refined based on clinical evolution and microbiological findings. Despite early aggressive therapy, three polytrauma patients developed recurrent infections, necessitating multiple surgical interventions. Upon stratification using the PJI-TNM system, the majority of infections (70%) were classified as N2 (mature biofilm formation), correlating with increased therapeutic challenges and a higher requirement for two-stage revision procedures. Among PJI cases, T2 (early loosening or moderate osteolysis) was the most frequent classification (45%), while 30% of cases presented with T3 (severe osteolysis, necessitating complex reconstruction). Regarding systemic status, 56% of patients were classified as M1 (moderate systemic disease), while 26% fell into the M2 category (severe systemic compromise, including multi-organ dysfunction). These stratifications provided a structured framework for treatment decisions, with higher T and N scores correlating with prolonged ICU stays and the need for extensive surgical interventions. Future studies with larger cohorts will allow further statistical validation of these observations.

### 3.2. Treatment Outcomes and Clinical Evolution

**Figure 2:**
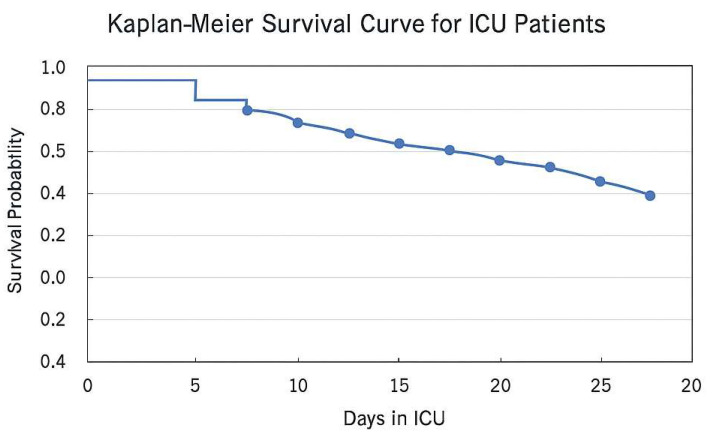
Kaplan–Meier Survival Curve for ICU Patients with Septic Orthopedic Infections which depicts the cumulative probability of survival for intensive care unit patients with septic orthopedic infections. The horizontal axis represents the number of days spent in the ICU, and the vertical axis depicts the likelihood of survival. As can be observed, the probability of survival declines gradually over time, with a notable decrease occurring after 10 days in the ICU. This illustrates the significant impact prolonged ICU stays have on patient outcomes. This analysis highlights the importance of early detection and aggressive treatment in cases of septic orthopedic infection to improve survival rates.

The mean time required to achieve infection control in the ICU was 13 days, with a range of 6 to 20 days. Infection resolution, as evidenced by the absence of positive microbiological cultures and the normalization of inflammatory markers, was achieved in 85,18% of cases. The patient outcomes were favorable, demonstrating a substantial enhancement in functional status and mobility, particularly in those who underwent two-stage revision operations. Of the 27 patients included in the study, three patients succumbed to their illness. Two deaths occurred within the first 30 days of ICU admission (30-day mortality rate: 7.4%) and one additional death occurred after 30 days but within the study period (overall mortality rate: 11%). A Kaplan–Meier survival analysis was conducted to enhance the visualization of survival outcomes for ICU patients with septic orthopedic infections. The presented curve illustrates the cumulative survival probability throughout the course of the patient’s stay in the ICU. The Kaplan–Meier survival curve (Figure 2) demonstrates a gradual reduction in survival probability over time, with a notable decline in survival rates following prolonged ICU admissions, particularly beyond 10 days. The curve serves to illustrate the significant impact of early intervention and intensive care on patient outcomes, particularly in cases where the patient presents with substantial comorbidities.

**Figure 3:**
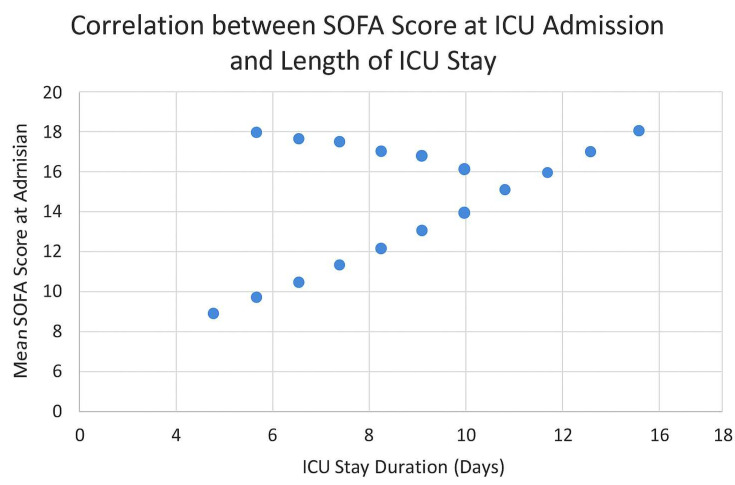
Correlation between SOFA Score at ICU Admission and Length of ICU Stay. The scatter plot illustrates the relationship between SOFA scores on ICU admission and the duration of ICU hospitalization. A positive correlation is observed, suggesting that patients with higher SOFA scores required longer ICU management. Each data point represents one patient (n = 27).

In order to further investigate the prognostic relevance of initial physiological severity in septic orthopedic patients, we analyzed the correlation between the Sequential Organ Failure Assessment (SOFA) score at ICU admission and the duration of critical care hospitalization. As illustrated in Figure 3, there was a discernible positive correlation between elevated SOFA scores and prolonged ICU stays. This relationship underscores the role of SOFA scoring not only as a bedside clinical tool for stratifying risk and guiding immediate therapeutic priorities but also as a potential early predictor of critical care burden and resource allocation. Patients presenting with SOFA scores ≥ 12 at admission were more likely to require extended ICU monitoring and mechanical ventilation, and had a slower inflammatory response normalization. Conversely, individuals with SOFA scores < 8 typically exhibited a more favorable trajectory, characterized by shorter ICU stays, faster stabilization, and fewer systemic complications. These findings are consistent with previously reported associations between multi-organ dysfunction severity and ICU outcomes in septic populations. The correlation visualized in Figure 3 complements the dynamic analysis of SOFA evolution within the first 48 h (see Figure 5), reinforcing the importance of continuous organ dysfunction monitoring during early ICU management. Together, these parameters may support the development of a prognostic algorithm that integrates both admission scores and early progression to anticipate clinical courses and optimize personalized care plans in high-risk orthopedic patients.

The evolution of the inflammatory syndrome was also assessed based on C-reactive protein (CRP), interleukin-6 (IL-6) plasma concentrations, and leukocyte counts, representing a key objective of our study. A significant reduction in the inflammatory syndrome was observed when comparing the inflammatory markers at the time of admission to the ICU with those at the end of treatment. The improvement values serve to demonstrate the efficacy of the therapeutic interventions in achieving remission of the infectious process. The error bars represent the standard deviation, which provides an indication of the variability observed within the patient groups (Table 3).

Glycemic control is of paramount importance in the management of patients with infectious processes. Patients with well-controlled HbA1c levels (less than 7%) exhibited accelerated CRP normalization (12.5 days), a reduced incidence of infection recurrence, and expedited bone healing (10.5 weeks). In contrast, patients with poorly controlled HbA1c levels (>8.5%) exhibited delayed CRP normalization (22.4 days), increased recurrence rates (30%), and slower bone healing (14.6 weeks) Tabel 3. These findings underscore the significance of rigorous glycemic management to enhance recovery and mitigate complications in patients with orthopedic infections. The influence of glycemic control, evaluated through HbA1c levels, was found to significantly impact inflammatory marker normalization and bone healing time. Table 4 highlights these associations, showing a clear trend between HbA1c levels and delayed CRP normalization and bone healing. These findings align with existing literature emphasizing the role of metabolic control in orthopedic infection outcomes. To further elucidate the clinical implications of glycemic control, statistical analyses were conducted comparing inflammatory marker normalization times and bone healing durations among HbA1c-defined patient groups (Group A: HbA1c < 7%, Group B: HbA1c 7–8.5%, Group C: HbA1c > 8.5%). ANOVA revealed significant differences among groups for CRP normalization times (F = 23.5, *p* < 0.001) and bone healing times (F = 18.2, *p* < 0.001). Specifically, patients in Group C exhibited significantly prolonged CRP normalization (mean: 22.4 days) compared to Group A (mean: 12.5 days; t = 5.62, *p* < 0.001) and significantly prolonged bone healing time (mean: 14.6 weeks) compared to Group A (mean: 10.5 weeks; t = 4.89, *p* < 0.001). Additionally, infection recurrence rates were statistically higher in Group C (30%) compared to Groups A (0%) and B (12.5%) (χ^2^ = 7.14, *p* = 0.028). These findings strongly underscore the critical role of stringent glycemic control as a determinant of improved clinical outcomes, emphasizing its necessity in septic orthopedic patient management protocols. Statistically significant differences were observed between the three groups in CRP normalization time (*p* < 0.001), bone healing duration (*p* = 0.002), and ICU stay length (*p* = 0.017), indicating a direct association between glycemic control and clinical recovery.

**Figure 4:**
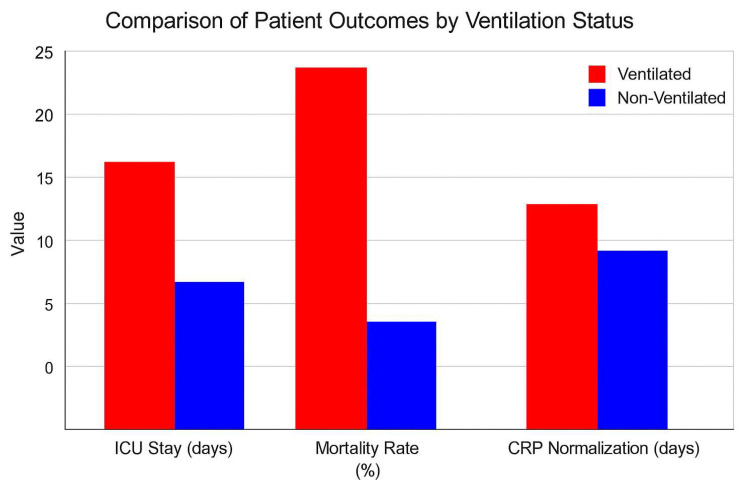
Clinical Outcomes Related to Mechanical Ventilation Requirement. Comparison between patients who required mechanical ventilation (MV) versus those who did not. The figure clearly demonstrates increased ICU length of stay (days) and mortality rate (%) among patients needing ventilatory support. These findings underscore mechanical ventilation as a critical prognostic factor in septic orthopedic ICU patients.

Mechanical ventilation is of pivotal importance in the management of septic orthopedic patients with respiratory compromise, frequently resulting from acute respiratory distress syndrome (ARDS) or sepsis-induced respiratory failure. In this study, 29.6% of patients required ventilatory support, which was associated with prolonged ICU stays (a mean of 14.2 days) and higher mortality rates (25%). These findings underscore the critical necessity for prompt respiratory support to ensure adequate oxygenation and tissue perfusion, particularly in patients with multi-organ dysfunction (Figure 4). Nevertheless, prolonged mechanical ventilation was also associated with an increased incidence of complications, including ventilator-associated pneumonia (VAP). This emphasizes the necessity of optimizing ventilation strategies and implementing robust protocols in order to mitigate the associated risks.

A number of studies published in the specialized literature attest to the fact that the prognosis of septic patients requiring renal supplementation therapy during their evolution is unfavorable. In our study, 25% of patients required CRRT. However, due to the limited number of patients, no significant differences in prognosis between those with AKI and those without AKI during their evolution were identified.

**Figure 5:**
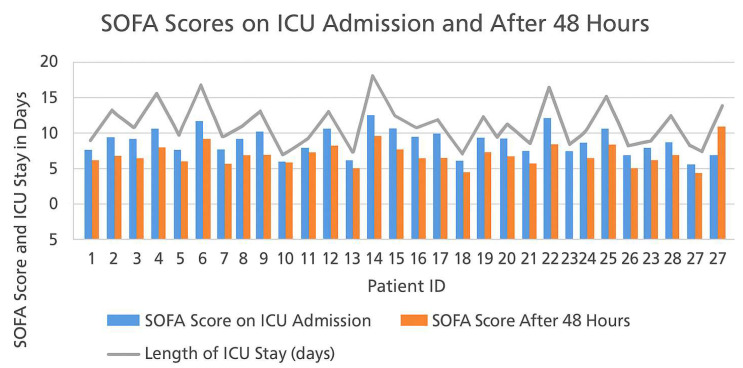
Sequential Organ Failure Assessment (SOFA) Scores at ICU Admission (blue) and 48 h (orange) for the entire cohort of 27 patients. The grey line represents the length of time spent in the ICU by each patient. Patients with elevated initial SOFA scores (e.g., patients 6, 14, 20) exhibited prolonged ICU admissions, indicating more severe organ dysfunction. Conversely, patients with lower SOFA scores upon admission and after 48 h (e.g., patients 3, 9, 12) exhibited reduced ICU durations and improved overall recovery. This evidence lends support to the use of the SOFA score as a prognostic indicator for the length of stay in the ICU and patient outcomes in cases of septic orthopedic disease.

The incidence of complications was 18% for septic shock, 30% for acute kidney damage (AKI), and 25% for ventilator-associated pneumonia (VAP). Despite the implementation of rigorous resuscitation and organ care measures, three patients succumbed to multi-organ failure during their ICU stay, resulting in an ICU mortality rate of 11%. Figure 5 illustrates the Sequential Organ Failure Assessment (SOFA) ratings documented at the time of ICU admission and 48 h thereafter for the 27 patients who participated in the study. The SOFA score is an essential prognostic instrument, employed to evaluate the level of organ dysfunction, and is frequently applied in septic patients to track their progression in the ICU. The image illustrates the correlation between SOFA scores and the length of time spent in the ICU, demonstrating the relationship between early organ dysfunction and patient outcomes.

Of the 27 patients, 18 underwent a two-stage revision procedure, which comprised initial debridement, insertion of antibiotic-loaded cement spacers, and subsequent replacement with a permanent prosthesis. The infection control rate among these patients was 89%, with 16 exhibiting no indications of infection recurrence at their last follow-up. Surgical stabilization of fractures was performed for the nine polytrauma patients within 24 to 48 h of admission to the ICU, followed by targeted antibacterial therapy. Despite the complexity of these cases, six patients achieved primary infection control and were discharged from the ICU with an improved clinical status. However, three patients experienced recurrent infections, necessitating further surgical procedures.

Several prognostic factors were evaluated in relation to infection resolution and mortality. Patients with a SOFA score ≥ 10 at ICU admission had a significantly prolonged ICU stay (mean 18.6 days vs. 10.4 days for SOFA < 10) and a higher mortality rate (28% vs. 4%, respectively). Glycemic control also played a critical role, as patients with HbA1c > 8.5% exhibited delayed inflammatory marker normalization (mean 22.4 days vs. 12.5 days in HbA1c < 7%) and a 30% recurrence rate of infection. Mechanical ventilation was required in 29.6% of cases, correlating with an increased length of ICU stay (mean 14.2 days) and higher mortality (25%). Although the limited sample size precluded multivariate statistical analysis, these trends suggest that initial SOFA scores, glycemic control, and the necessity for ventilatory support are key determinants of patient outcomes.

### 3.3. Highlighted Clinical Cases

Two cases were selected for detailed clinical illustration based on their complexity, severity, and representative nature. Both patients exhibited high PJI-TNM classification scores, multiple comorbidities, and required prolonged ICU stays with multidisciplinary interventions. These cases were deemed clinically illustrative due to the challenges posed in terms of infection control, metabolic stabilization, and orthopedic reconstruction. The selection aimed to highlight the decision-making rationale, therapeutic sequencing, and outcome variability in high-risk septic orthopedic patients.

**Figure 6:**
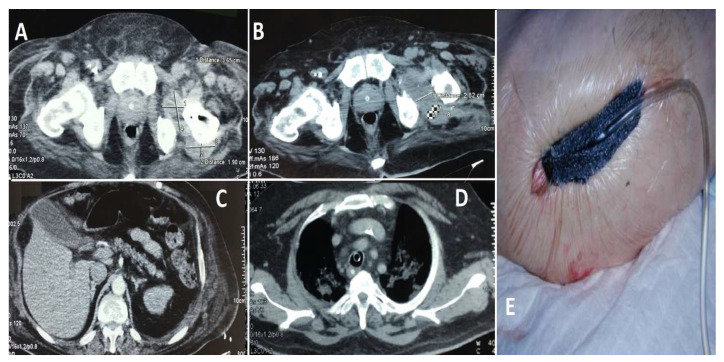
Images (**A**,**B**): The presence of a notable fluid collection in the hip region suggests the possibility of a periprosthetic infection. The presence of this accumulation was a crucial factor in the diagnosis of the infection. Image (**C**): A splenic lesion is observed, necessitating a splenectomy due to spontaneous rupture resulting from infectious complications. Image (**D**): The presence of valve vegetations on the aortic valve necessitates the performance of a valvuloplasty. The vegetations were identified through the utilization of transesophageal echocardiography, a diagnostic technique employed to assess the patient’s condition. Image (**E**) depicts the postoperative status of the hip, which has been fitted with a negative pressure drainage device to facilitate wound healing and reduce the accumulation of contaminated fluid.

Case 1 (see Figure 6): A 67-year-old male with multiple comorbidities, including chronic renal failure, morbid obesity, diabetes mellitus, chronic heart failure, two coronary stents, and atrial fibrillation necessitating long-term anticoagulation therapy, presented with a septic infection of a hip prosthesis implanted approximately 10 years prior. The patient’s substantial comorbidities had a significant impact on both the surgical procedure and subsequent care. During his hospitalization, the patient displayed electrocardiogram (ECG) alterations suggestive of an ST-elevation myocardial infarction (STEMI) in the inferolateral region. Subsequent diagnostic assessments, including transesophageal echocardiography, identified the presence of vegetation on the aortic valve, indicative of endocarditis. The initial decision was to defer the planned cardiovascular surgery due to the patient’s rapidly deteriorating general condition and precarious health status. Instead, broad-spectrum antibiotics were provided. Following a two-week course of antibiotic treatment, the patient experienced a spontaneous splenic rupture, necessitating an emergency splenectomy; the patient’s condition continued to deteriorate. A subsequent transesophageal ultrasound revealed further expansion of the valvular vegetations, necessitating immediate valvuloplasty. The procedure was completed successfully, and the patient was initiated on a robust anticoagulant regimen postoperatively. A computed tomography (CT) scan of the hip revealed the presence of periprosthetic collections, thereby confirming the persistence of the local infection. A revision arthroplasty was performed, during which a cemented prosthesis was inserted to replace the contaminated hardware. Despite these interventions, the patient’s general health continued to deteriorate due to the complexity of his medical condition and the persistence of the infection. Despite comprehensive antibiotic therapy, surgical procedures, and intensive care support, the patient experienced multi-organ failure and ultimately succumbed to their illness after approximately six weeks of treatment.

**Figure 7:**
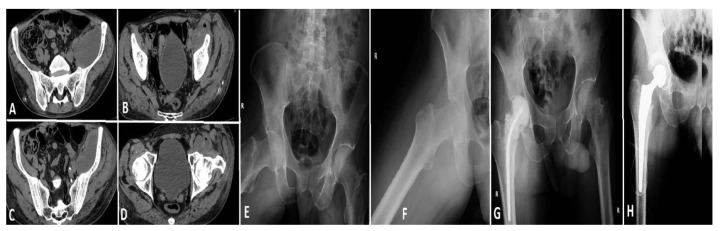
Depicts three images, labeled (**A**–**C**), which illustrate a significant pelvic abscess that extends along the iliopsoas muscle and reaches the femoral head. This collection indicates a profound infection, with the potential to affect both the muscle and the surrounding joint components. Image (**D**) illustrates the abscess encircling the femoral head, thereby emphasizing the severity of the infection in this vital anatomical area. Images (**E**,**F**) illustrate the conventional anteroposterior and lateral radiographs of the hip joint, respectively. Despite the X-rays failing to provide sufficient information to elucidate the diagnosis, the patient’s ongoing and intense discomfort necessitated additional imaging via a CT scan, which ultimately disclosed the full extent of the illness. Image (**G**) depicts the surgical debridement of the infected joint focus, accompanied by the insertion of an antibiotic-impregnated cement spacer. The objective of this provisional implant is to manage the infection while ensuring the mechanical stability of the joint. In Image (**H**), the hip arthroplasty is depicted, conducted approximately six months following the initial surgical procedure, when the infection was successfully managed, thereby allowing for the placement of the definitive prosthesis.

Case 2 (see Figure 7): A 54-year-old male, despite being younger than the typical age for this condition, exhibited a considerable number of comorbidities, including a history of myocardial infarction, diabetes mellitus, persistent smoking, alcohol misuse, and a cachectic appearance. The patient was a pedestrian involved in a road traffic accident three months prior, yet did not seek medical attention at the time. The patient presented at the hospital three months after the incident with severe left hip pain. Imaging and clinical evaluation indicated the presence of purulent discharge from the left hip, while laboratory tests demonstrated a notable elevation in inflammatory markers. The patient was transferred to the ICU with sepsis and underwent a hip osteotomy, followed by the insertion of an antibiotic-impregnated spacer. Following intensive antimicrobial treatment and supportive care in the ICU, the patient’s condition showed signs of improvement. However, due to the presence of chronic health issues and an elevated risk of infection recurrence, the long-term prognosis remained uncertain.

## 4. Discussion

This study offers significant insights into the complex management of septic orthopedic infections, particularly in patients with substantial comorbidities and those requiring ICU interventions. Our findings highlight the challenges associated with the management of periprosthetic joint infections and trauma-related sepsis, particularly in elderly patients, those with obesity, or those with pre-existing chronic conditions such as diabetes mellitus, renal failure, and cardiovascular disease [1,22].

From a clinical perspective, our findings reinforce the critical need for early identification and referral of high-risk PJI cases to specialized centers with intensive care capabilities. The observed association between SOFA scores, ICU duration, and outcomes emphasizes that delayed transfer or conservative management in non-specialized units may compromise prognosis. Patients exhibiting signs of systemic decompensation—particularly those with elevated inflammatory markers, advanced PJI-TNM stages, or poorly controlled metabolic profiles—should prompt immediate multidisciplinary evaluation and potential ICU admission. The intensive use of ICU resources, including mechanical ventilation, vasopressor support, and prolonged antibiotic stewardship, was justified by the high infection resolution rate (85.2%) and the relatively low ICU mortality (11%). However, this benefit must be balanced against healthcare resource constraints, particularly in systems with limited access to tertiary-level critical care. Therefore, structured referral pathways, standardized PJI-TNM-based triage protocols, and integration of early warning scores (e.g., SOFA, qSOFA) in orthopedic infection units could enhance decision-making, optimize resource utilization, and improve outcomes for septic orthopedic patients. These insights suggest that beyond infection control alone, systemic stabilization through early ICU intervention plays a pivotal role in long-term functional recovery and survival, particularly in polymorbid or diabetic populations. Future prospective studies should explore cost-effectiveness models for ICU-based orthopedic sepsis management, with particular attention to identifying thresholds for early escalation of care.

A notable parallel can be drawn between the elderly population with hip fractures and heart failure, as evidenced by prior studies. These individuals frequently exhibit diminished immune function and are at an elevated risk of infection due to a confluence of age-related immunological deterioration and the presence of multiple comorbidities. The population demonstrated comparable susceptibility, with elevated neutrophil counts indicative of the systemic inflammatory response syndrome (SIRS), resulting in tissue damage and elevated mortality risk, particularly among individuals with heart failure. Our investigation revealed that patients with diminished immunological responses, including those with diabetes and renal failure, had a markedly elevated incidence of complications. This finding is consistent with evidence indicating that impaired immune function worsens infection-related outcomes in older and comorbid individuals. The 70% rate of chronic infections observed in our cohort aligns with recent literature, where reported rates range from 60% to 75%, depending on classification methods and patient population characteristics. This finding reflects the high-risk profile of our patients, who frequently present with multiple comorbidities and compromised immune systems [10,23,24].

The incorporation of ICU care in the treatment of septic orthopedic patients is crucial, especially for trauma and polytrauma cases. The utilization of the Sequential Organ Failure Assessment (SOFA) and its expedited variant, Quick SOFA (qSOFA), has been demonstrated to be an effective method of identifying individuals who are susceptible to multi-organ failure and adverse outcomes. In our cohort, individuals with qSOFA scores of 2 or above had prolonged ICU admissions and elevated mortality rates. These findings are in accordance with the current literature, which indicates that elevated qSOFA scores are associated with poorer prognoses in patients with sepsis. The principles of damage control orthopedics (DCO) are of paramount importance in the management of polytrauma patients, particularly those with septic illnesses resulting from multiple fractures and soft tissue injuries. In such cases, our strategy involved the prompt performance of surgical procedures, the stabilization of fractures, and the implementation of rigorous infection management protocols. This approach is consistent with prior research that underscores the importance of limiting surgical stress while prioritizing infection management, with the aim of reducing mortality and morbidity. Furthermore, it is crucial to recognize that trauma patients with open fractures frequently present with a polluted condition, rendering them particularly susceptible to sepsis [5,12,25,26].

The subsequent table (Table 5) presents a comprehensive synthesis of pivotal findings from recent studies examining the management of septic orthopedic infections within the context of ICU interventions. This table elucidates significant prognostic factors, such as the predictive value of inflammatory markers and comorbidities, alongside the critical role of ICU protocols in influencing key clinical outcomes, including mortality rates, CRP normalization, and the incidence of complications. The collated data highlight the complex interplay between patient-specific variables, such as diabetes and obesity, and the necessity for tailored approaches to optimize care. These findings align with the core principles of our investigation, particularly the importance of a multidisciplinary approach, the expedient identification of high-risk patients through tools like qSOFA, and the precise implementation of infection control and organ-support protocols. Furthermore, the evidence presented reinforces the indispensable role of ICU management in mitigating adverse outcomes and advancing recovery trajectories in this vulnerable patient population. The observed 30-day mortality rate (7.4%) and overall mortality rate (11%) are consistent with findings from similar cohorts described in the literature. This difference emphasizes the importance of early intervention in critically ill septic orthopedic patients to reduce early mortality risks [20,27].

The deleterious effect of obesity on the prognosis of polytrauma patients is well-documented. A substantial body of research has demonstrated that obesity is associated with prolonged hospital and intensive care unit admissions and an increased incidence of complications. Our study corroborates these findings, as obese individuals exhibited a markedly extended duration of stay in the ICU and had a higher propensity to develop comorbidities, including acute renal failure and wound infections. The prolonged period of mechanical ventilation observed in obese individuals aligns with prior findings indicating that obesity exacerbates respiratory mechanics and heightens the necessity for prolonged mechanical support. The investigation revealed that obesity significantly affected ICU length of stay; however, there was no statistically significant increase in the duration of mechanical ventilation. This aligns with prior studies that present mixed findings on this outcome [1,7,29].

Our findings indicate a significant association between HbA1c levels and both CRP normalization time and bone healing duration. Patients with poorly controlled HbA1c (>8.5%) exhibited delayed inflammatory marker normalization and slower bone healing, reinforcing the critical importance of metabolic control in managing septic orthopedic infections. These observations align with existing literature emphasizing the detrimental effects of poor glycemic control on infection resolution and healing. Future studies could further explore the mechanistic links between glycemic variability and immune response in septic orthopedic patients. The management of blood glucose in patients with sepsis has been a topic of considerable debate and controversy [25,26,27,28,29].

The findings of our investigation align with those of prior meta-analyses, indicating that both intensive and liberal glucose control regimens yield comparable outcomes with respect to mortality and ICU length of stay. However, intensive glucose control is associated with an elevated risk of hypoglycemia. The distinction between polytrauma and PJI is essential for understanding the varied clinical trajectories of septic orthopedic patients. While these groups were analyzed separately, occasional overlaps were observed, particularly in trauma patients who developed prosthetic infections post-surgery. Future studies with larger cohorts might further explore these intersections. Additionally, the observed relationship between HbA1c levels and inflammatory marker normalization emphasizes the importance of metabolic control in improving infection outcomes. Although the PJI-TNM classification system was employed in our study to guide treatment decisions, detailed subgroup analyses based on TNM stages were not included in the current results. Incorporating these findings in future analyses may provide deeper insights into risk stratification and personalized care pathways [30,31,32,33].

The distinction between polytrauma-related sepsis and PJI remains clinically significant, particularly given the occasional overlap between these categories. While our study primarily categorized patients based on their dominant clinical presentation, future studies could provide a more nuanced analysis of overlapping cases. Additionally, our findings reinforce the importance of glycemic control, as reflected by HbA1c levels, in predicting CRP normalization and bone healing time. Finally, while the PJI-TNM classification system was employed in treatment planning, its full potential for risk stratification and guiding therapeutic decisions was limited by the sample size. Future research should aim to integrate these variables more systematically to refine treatment algorithms [14,15,34,35].

A noteworthy finding of our study is the elevated incidence of complications observed in patients with multiple comorbidities. The presence of diabetes, obesity, and cardiovascular disease significantly hindered infection management, resulting in prolonged treatment durations. These findings are consistent with those of other researchers who have indicated that comorbidities, including renal failure and obesity, have an adverse effect on prognosis and elevate the chance of multi-organ failure. Our findings are in accordance with existing evidence which indicates that prompt and assertive intervention in high-risk patients is necessary in order to prevent the advancement of sepsis and to reduce the occurrence of failure to rescue (FTR), whereby complications such as pneumonia, respiratory failure, and arrhythmias may result in mortality [18,36].

The application of the PJI-TNM classification in this study provided valuable insights into infection complexity and treatment stratification. While the PJI-TNM classification system was employed in our study primarily as a stratification tool, we acknowledge the necessity for a deeper exploration of its prognostic and therapeutic implications. Our findings suggest preliminary associations between higher T and N stages (reflecting severe implant loosening, bone loss, and mature biofilm presence) and the necessity for more aggressive surgical interventions, prolonged intensive care unit stays, and increased infection recurrence rates. Similarly, the presence of significant comorbidities (higher M scores) correlated with extended recovery periods and a higher incidence of complications, reinforcing the utility of this classification as a predictor of adverse outcomes. Nevertheless, due to the limited sample size, definitive subgroup analyses exploring the nuanced interactions between the individual T, N, and M stages and specific therapeutic outcomes were beyond our current statistical capabilities. Future prospective studies, particularly with larger cohorts, should systematically evaluate the predictive accuracy of each classification dimension (T, N, M) regarding both clinical decision-making and patient prognosis. Such analyses would enable a more personalized therapeutic approach, potentially enhancing infection control, reducing morbidity, and optimizing recovery trajectories in high-risk orthopedic patients. The association between higher SOFA scores and increased mortality is consistent with broader critical care literature, reinforcing its utility as a risk stratification tool in orthopedic sepsis. The impact of glycemic control on infection recurrence and inflammatory resolution is also well-documented, further supporting the need for stringent metabolic management in these patients. Additionally, the correlation between mechanical ventilation and prolonged ICU stay underscores the importance of early respiratory optimization in patients with sepsis-induced organ dysfunction. While a more robust statistical approach would be beneficial in a larger cohort, these preliminary findings highlight clinically relevant trends that warrant further investigation in future studies.

We acknowledge that the current study possesses intrinsic limitations related primarily to its retrospective nature and the relatively small cohort size (n = 27). Although the specificity of our inclusion criteria—patients with severe orthopedic sepsis necessitating critical care interventions—has ensured a highly focused and clinically relevant population, the modest sample inevitably constrains the statistical power of our analysis. Consequently, the ability to generalize these findings may be restricted. Additionally, nuanced subgroup analyses based on the PJI-TNM classification could not be statistically validated fully, further underscoring the need for larger patient cohorts. Another limitation lies in the heterogeneity of the underlying pathology, as the study included both patients with periprosthetic joint infections (PJI) and those with polytrauma-related sepsis, which may have introduced variability in disease trajectory and therapeutic response. Although efforts were made to analyze outcomes based on glycemic control, comorbidity burden, and ICU metrics, multivariate models and propensity score adjustments could not be applied due to sample size limitations. Furthermore, certain clinical parameters, such as body mass index (BMI), were not uniformly documented, and long-term follow-up data, including reinfection and functional recovery, were unavailable for some patients. While the PJI-TNM classification was applied retrospectively to PJI cases, its role in clinical stratification requires further validation. The external studies listed in Table 5 were not selected through a formal systematic review but were chosen based on thematic relevance to the core clinical aspects encountered in our cohort. Future multicenter studies with greater sample sizes are warranted to validate our preliminary findings robustly, refine risk stratification methods, and optimize tailored treatment protocols. Collaboration among institutions will facilitate comprehensive assessments, enhance external validity, and strengthen the overall evidence base guiding intensive care management of septic orthopedic patients.

## 5. Conclusions

This study highlights the importance of ICU-level management in improving outcomes for septic orthopedic patients, particularly those with periprosthetic joint infections and polytrauma-related sepsis. Although constrained by the retrospective design and modest sample size, our findings suggest that early ICU admission, individualized management strategies, and careful assessment of comorbidity burden may positively influence recovery trajectories. The application of the PJI-TNM classification provided a useful framework for understanding disease complexity in PJI cases, especially in patients with mature biofilms (M1) and severe systemic comorbidities (N2). While formal validation is still needed, this system may support future risk stratification in critical care orthopedic pathways. Further prospective, multicenter studies are required to confirm these preliminary insights and to help develop standardized, globally applicable protocols for the prevention and intensive management of orthopedic implant-associated infections. Tailored multidisciplinary approaches remain essential to improving long-term outcomes in this high-risk patient population.

## Figures and Tables

**Table 1:** The PJI-TNM classification system—stratifies periprosthetic joint infections based on T (Tissue and Implant Status), N (Necrotic and Biofilm Formation), and M (Medical Condition and Comorbidities). This framework evaluates implant stability (T), biofilm presence (N), and patient comorbidities (M) to guide treatment decisions. Higher T and N stages indicate increased infection chronicity and biofilm maturity, while M stages reflect systemic disease burden, influencing prognosis and surgical approach.

Category	Classification	Description
T (Tissue and Implant Status)	T1	Stable implant, no significant bone loss
	T2	Early loosening or osteolysis with moderate bone loss
	T3	Severe osteolysis, implant failure requiring complex reconstruction
N (Necrotic and Biofilm Formation)	N0	No biofilm detected
	N1	Immature biofilm with low bacterial load (infection within 4 weeks)
	N2	Mature biofilm confirmed microbiologically or histologically (>4 weeks, chronic infection)
M (Medical Condition and Comorbidities)	M0	No significant comorbidities
	M1	Moderate systemic disease (e.g., controlled diabetes, hypertension)
	M2	Severe comorbidities (e.g., immunosuppression, multiple organ dysfunction)

**Table 2:** Patient Demographics and Infection Characteristics provide a summary of the characteristics of each patient, including the type of infection, the presence of comorbidities, and the clinical outcomes. The following table demonstrates the interrelationship between patient health status, infection severity, and therapeutic modalities. MRSA—Methicillin-Resistant Staphylococcus aureus, MSSA—Methicillin-Sensitive Staphylococcus aureus, CVD—Cardiovascular Disease, ARF—Acute Renal Failure.

Patient ID	Age (years)	Gender	Infection Type	Pathogen	Comorbidities
1	72	Male	PJI (T2/N2/M1)	MRSA	Diabetes, CVD
2	68	Female	Polytrauma	Mixed Flora	Renal Failure
3	79	Male	PJI (T3/N1/M1)	Candida albicans	Diabetes
4	55	Female	PJI (T2/N1/M1)	MRSA	Obesity
5	61	Male	PJI (T2/N2/M1)	MRSA	CVD
6	83	Female	Polytrauma	Mixed Flora	Obesity, ARF
7	58	Male	PJI (T1/N2/M0)	Staphylococcus epidermidis	Diabetes, CVD
8	64	Female	Polytrauma	Mixed Flora	Obesity, Diabetes
9	75	Male	PJI (T1/N1/M0)	MSSA	CVD
10	49	Male	Polytrauma	Mixed Flora	Renal Failure
11	77	Female	PJI (T2/N1/M1)	MRSA	Diabetes
12	62	Male	PJI (T1/N2/M0)	Staphylococcus epidermidis	CVD
13	69	Female	PJI (T2/N2/M0)	MSSA	Obesity, Diabetes
14	54	Male	Polytrauma	Mixed Flora	ARF
15	63	Female	PJI (T2/N2/M0)	MSSA	Diabetes, CVD
16	67	Male	PJI (T2/N1/M0)	MSSA	CVD
17	73	Male	Polytrauma	Mixed Flora	ARF
18	51	Male	Polytrauma	Mixed Flora	Obesity
19	76	Female	PJI (T2/N1/M0)	Staphylococcus epidermidis	Diabetes
20	68	Female	Polytrauma	Mixed Flora	CVD
21	59	Male	PJI (T1/N1/M0)	MSSA	Diabetes
22	42	Male	Polytrauma	Mixed Flora	Obesity, Diabetes
23	74	Female	PJI (T3/N2/M1)	MRSA	Diabetes
24	70	Female	PJI (T1/N1/M0)	Staphylococcus epidermidis	CVD
25	71	Male	PJI (T2/N1/M0)	MSSA	Diabetes
26	53	Female	Polytrauma	Mixed Flora	Obesity, Diabetes
27	66	Male	PJI (T3/N2/M1)	MRSA	ARF

**Table 3:** Evolution and Improvement of Systemic Inflammatory Markers from Admission to Discharge. Improvement was calculated as the difference between admission and discharge values. Mean values are presented with standard deviation (SD). Normal ranges: CRP < 5 mg/L; IL-6 < 7 pg/mL; WBC 4–10 × 10⁹/L.

Marker	Admission (Mean ± SD)	Discharge (Mean ± SD)	Improvement (Mean ± SD)
CRP (mg/L)	214.08 ± 9.62	12.67 ± 1.37	201.41 ± 8.68
IL-6 (pg/mL)	181.75 ± 7.59	15.33 ± 1.40	166.42 ± 6.47
WBC (10^9^/L)	15.48 ± 0.60	7.16 ± 0.32	8.32 ± 0.31

**Table 4:** Impact of Glycemic Control on Clinical Outcomes in Patients with Infectious Processes.

Group	Mean HbA1c (%)	Glycemic Range (mg/dL)	CRP Normalization (Days)	Infection Recurrence (%)	Bone Healing Time (Weeks)	Mean Glycemic Values (mg/dL)	Time to Bone Remodeling (Weeks)
Group A (HbA1c < 7%)	6.4	110–140	12.5 ± 1.5 †	0	10.5 ± 1.2 †	120	10.5
Group B (HbA1c 7–8.5%)	7.7	141–180	16.2 ± 1.7 *	12.5% *	12.3 ± 1.3 *	160	12
Group C (HbA1c > 8.5%)	9.1	181–340	22.4 ± 2.0 †	30% †	14.6 ± 1.5 †	220	14.5

Notes: CRP normalization: ANOVA, F = 23.5, *p* < 0.001; Pairwise A vs. C: t = 5.62, *p* < 0.001; Bone healing time: ANOVA, F = 18.2, *p* < 0.001; Pairwise A vs. C: t = 4.89, *p* < 0.001; Infection recurrence rate: χ^2^ = 7.14, *p* = 0.028; Legend: † Statistically significant difference (*p* < 0.001) between Group A and Group C; * Indicates statistically significant difference between Group B and Group C (*p* < 0.05); Intermediate values significantly different compared to Group C (*p* < 0.05), but less pronounced.

**Table 5:** Clinical Insights and Prognostic Factors in ICU Management of Septic Orthopedic Infections.

First Author (Year)	Study Focus	Key Findings	Sample Size	Relevance to Article
Lu et al. (2023) [13]	ICU mortality in elderly with orthopedic infections	Neutrophil percentage and INR identified as significant predictors of 30-day mortality.	641	Highlights the importance of early monitoring.
He et al. (2022) [26]	ICU-acquired infections in septic patients	Mechanical ventilation increased infection risks; tracheostomy linked to prolonged ICU stays.	16,808	Emphasizes ventilatory support’s role in outcomes.
Wang et al. (2023) [5]	Pathogen profiles in orthopedic infections	S. aureus dominated; antibiotic resistance trends observed, complicating treatment strategies.	2821	Reinforces pathogen-driven infection management.
Yang et al. (2023) [27]	Predicting sepsis onset in ICU patients	Machine learning models (Random Forest, XGBoost) achieved high predictive accuracy for early sepsis detection, improving outcomes through timely interventions.	4,314,145	Highlights innovative tools for early identification of sepsis and their role in improving ICU patient management.
Wildeman et al. (2021) [14]	Long-term PJI outcomes	1-year mortality at 13.6%, increasing to 25.6% at 5 years; comorbidities worsened long-term survival.	200	Stresses the importance of addressing comorbidities.
Yuan et al. (2021) [10]	Prognostic factors in bedridden elderly with hip fractures and infections	Prolonged bedridden time significantly increased mortality risk, highlighting glucocorticoid use and malnutrition as major risk factors.	101	Reinforces the role of early intervention and nutrition in ICU outcomes.
Li et al. (2024) [28]	Biomarker panel for sepsis prediction in trauma patients	Combination of CRP, PCT, and SAA (bioscore) showed superior diagnostic accuracy for sepsis compared to individual biomarkers.	100	Highlights the utility of biomarker panels in improving early sepsis detection and patient stratification.

## Data Availability

The data that support the findings of this study are available from the corresponding author upon reasonable request. Due to institutional regulations, the dataset is not publicly accessible.

## Abbreviations

The following abbreviations are used in this manuscript:

ICU	Intensive Care Unit
PJI	Periprosthetic Joint Infection
PJIs	Periprosthetic Joint Infections
TNM	Tissue, Necrosis, and Medical (in PJI-TNM classification)
MRSA	Methicillin-Resistant Staphylococcus Aureus
MSSA	Methicillin-Sensitive Staphylococcus Aureus
CVD	Cardiovascular Disease
ARDS	Acute Respiratory Distress Syndrome
CRRT	Continuous Renal Replacement Therapy
SOFA	Sequential Organ Failure Assessment
qSOFA	Quick Sequential Organ Failure Assessment
AKI	Acute Kidney Injury
HbA1c	Hemoglobin A1c
VAP	Ventilator-Associated Pneumonia
FTR	Failure to Rescue
STEMI	ST-Elevation Myocardial Infarction
ECG	Electrocardiogram
CT	Computed Tomography
SIRS	Systemic Inflammatory Response Syndrome
CARS	Compensatory Anti-Inflammatory Response Syndrome
CMV	Cytomegalovirus
CRP	C-Reactive Protein
IL-6	Interleukin-6
WBC	White Blood Cell Count
GDPR	General Data Protection Regulation
DCO	Damage Control Orthopedics
PCT	Procalcitonin
SAA	Serum Amyloid A
